# Supplementary material for: Boosting NAD+ with a small molecule that activates NAMPT
Source: Nat Commun. 2019 Jul 19;10:3241. doi: 10.1038/s41467-019-11078-z (PMC6642140; doi:10.1038/s41467-019-11078-z)
Supplement: Supplementary file 1 — Supplementary Information [file 41467_2019_11078_MOESM1_ESM.docx]

**Supplementary Information**

**Boosting NAD^+^ with a Small Molecule that Activates NAMPT**

**S.J. Gardell et al**

**Supplementary Figure 1**

a.

b.

**Impact of SBI-136892 (HTS hit) on NAMPT.** a. SBI-136892 increases the NAMPT thermal melting temperature (∆Tm). NAMPT was treated with DMSO (control) or varying concentrations of SBI-136892 in the presence of Sypro Orange dye and ATP for 15 min at room temperature. The temperature was ramped up (0.15° C sec^-1^) from 25 to 95°C and the Tm was calculated. Data are expressed as means ± s.d.; n=6 (cumulative data from 3 independent runs with duplicate samples at each SBI-136892 concentration). b. SBI-136892 stimulates NAMPT-mediated NMN production. NAMPT (16 nM) was incubated with NAM (5 μM), PRPP (6.25 μM), ATP (0.12 mM) and varying concentrations of SBI-136892 for 2 h at room temperature. NMN was assayed using the fluorescence assay. Each data point shows the mean values derived from 2 replicates. Source data are provided as a Source Data file.

**Supplementary Figure 2**

**
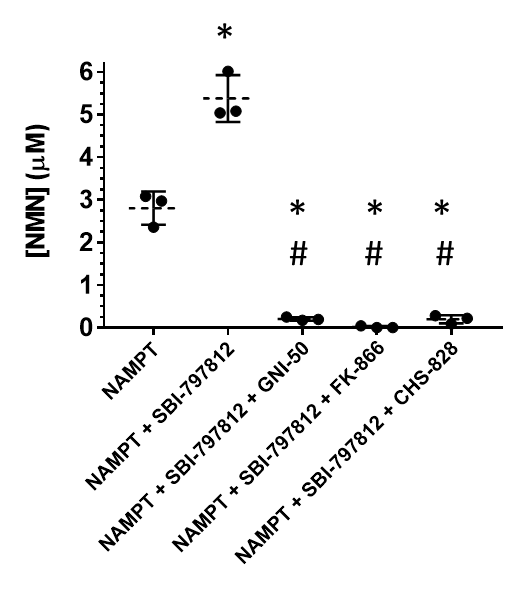
**

**Activation of NAMPT-mediated NMN production by SBI-797812 was blocked by NAMPT inhibitors**. NMN production was measured in the presence of SBI-797812 (2 μM), GNI-50 (5 μM), FK-866 (1 μM) or CHS-828 (1 μM) where indicated. Data is expressed as means ± s.d.; n=3. *, p<0.0001 compared to NAMPT; #, p<0.0001 compared to (NAMPT+SBI-797812). One-way ANOVA with Tukey’s multiple comparisons test was used. Source data are provided as a Source Data file.

**Supplementary Figure 3**

a.

b.

*

*

**P7C3-S243 and P7C3-A2 are not direct NAMPT activators**. (a) NAMPT inhibitors, CHS-828 and FK-866, but not P7C3-S243 stabilized purified human NAMPT in the PTS assay. The thermal melting temperature (Tm, ^o^C) of NAMPT was determined with increasing concentrations of CHS-828 (▲), FK-866 (■) and P7C3-S243 (●). (b) SBI-797812 but not P7C2-A2 or P7C3-S243 stimulated NMN production by human NAMPT in the presence of NAM, PRPP. and ATP. All data are expressed as means ± s.d.; n=3. *, p=0.0001 vs. NAMPT. One-way ANOVA performed on raw data; Dunnett’s multiple comparisons test was used. Source data are provided as a Source Data file.

**Supplementary Figure 4**

Control

SBI-797812

*

#

#

#

**Impact of SBI-797812 on NMN and ADP production.** Human NAMPT (30 nM) was incubated with NAM (10 μM), PRPP (50 μM), ATP (2 mM) in the absence or presence of SBI-797812 (2 μM) for 30 min at 37°C. Samples were quenched with equivolume 1M PCA and assayed for NMN and ADP by LC-MS/MS. Data are expressed as means ± s.d.; n=8. *, p=0.01; #, p<0.001. 1 way ANOVA with Tukey’s multiple comparisons test was used. Source data are provided as a Source Data file.

**Supplementary Figure 5**

**
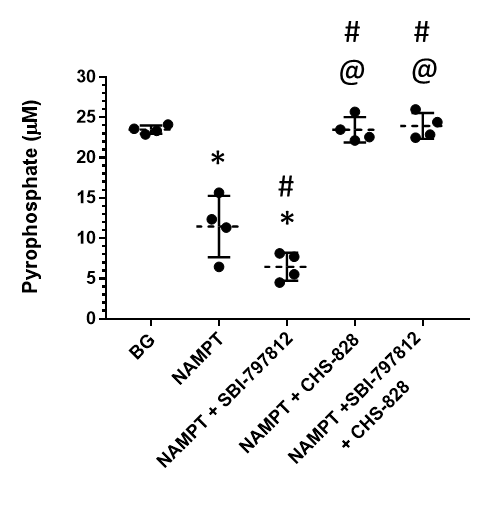
**

**Consumption of PP by NAMPT is blocked by CHS-828**. NAMPT (100 nM) was incubated in TMD buffer containing ATP (2 mM) and PP (20 μM) for 2 h at 37°C. Where indicated, SBI-797812 (5 μM) and/or CHS-828 (2 μM) were also included. PP production was determined with the colorimetric assay. Data are expressed as means ± s.d.; n=4. *, p< 0.001 vs. BG; #, p= 0.03 vs. NAMPT; @, p<0.001 vs. NAMPT; $, p<0.001 vs. NAMPT + SBI-797812. 1 way ANOVA with Tukey’s multiple comparisons test was used. Source data are provided as a Source Data file.

**Supplementary Figure 6**

 **a.**

**b. c.**

**SBI-797812 stimulated the facultative ATPase activity of NAMPT.** Human NAMPT (200 nM) was incubated with ATP (2 mM) at 37°C for increasing times in the absence (○) or presence of (●) SBI-797812 (2 μM). Samples were removed at the indicated times and measured repeatedly for ADP (a), Pi (b) and Ap4 (c). ADP and Ap4 were assayed by LC-MS/MS; Pi was assayed by the colorimetric assay. Linear regression for product formation over time is shown. Source data are provided as a Source Data file.

**Supplementary Figure 7**

NAMPT

NAMPT + ATP (Heat 95C)

NAMPT + ATP +NMN

NAMPT + ATP + PP

NAMPT + ATP

NAMPT + ATP + NAM

NAMPT + ATP + PRPP

NAMPT + ATP + NAM + PRPP


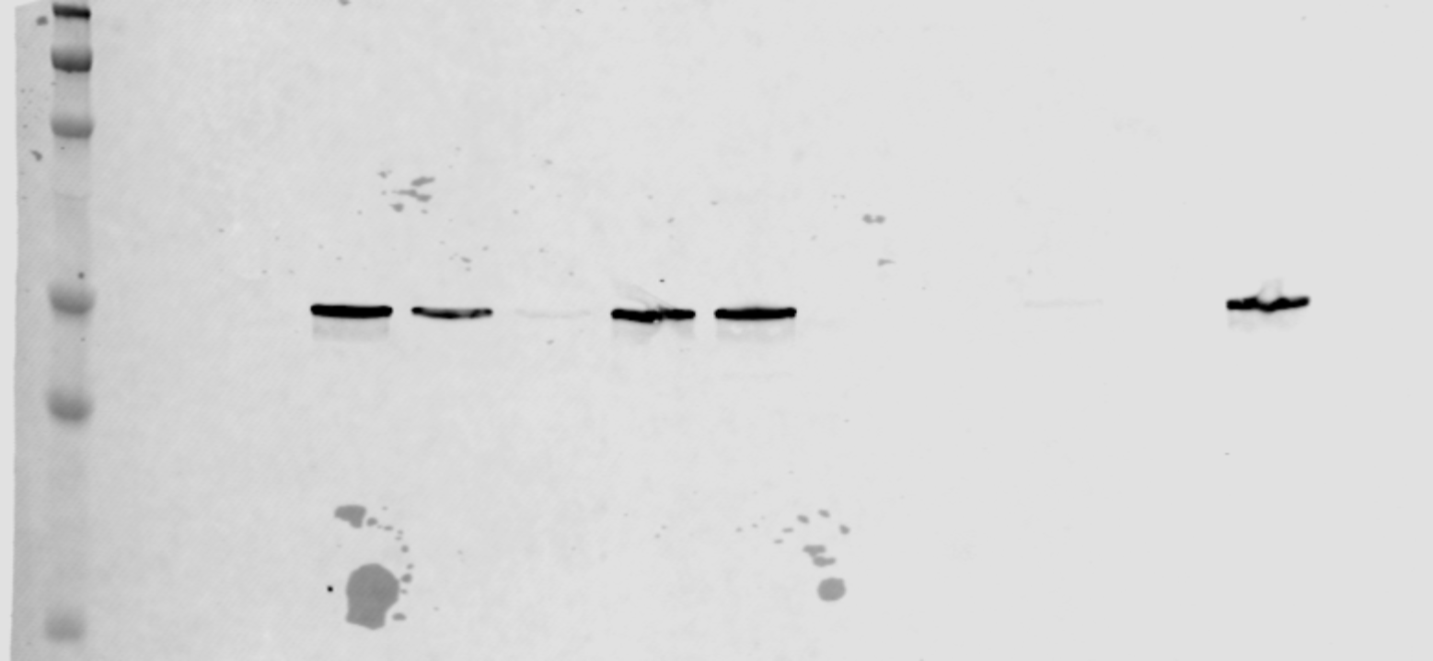

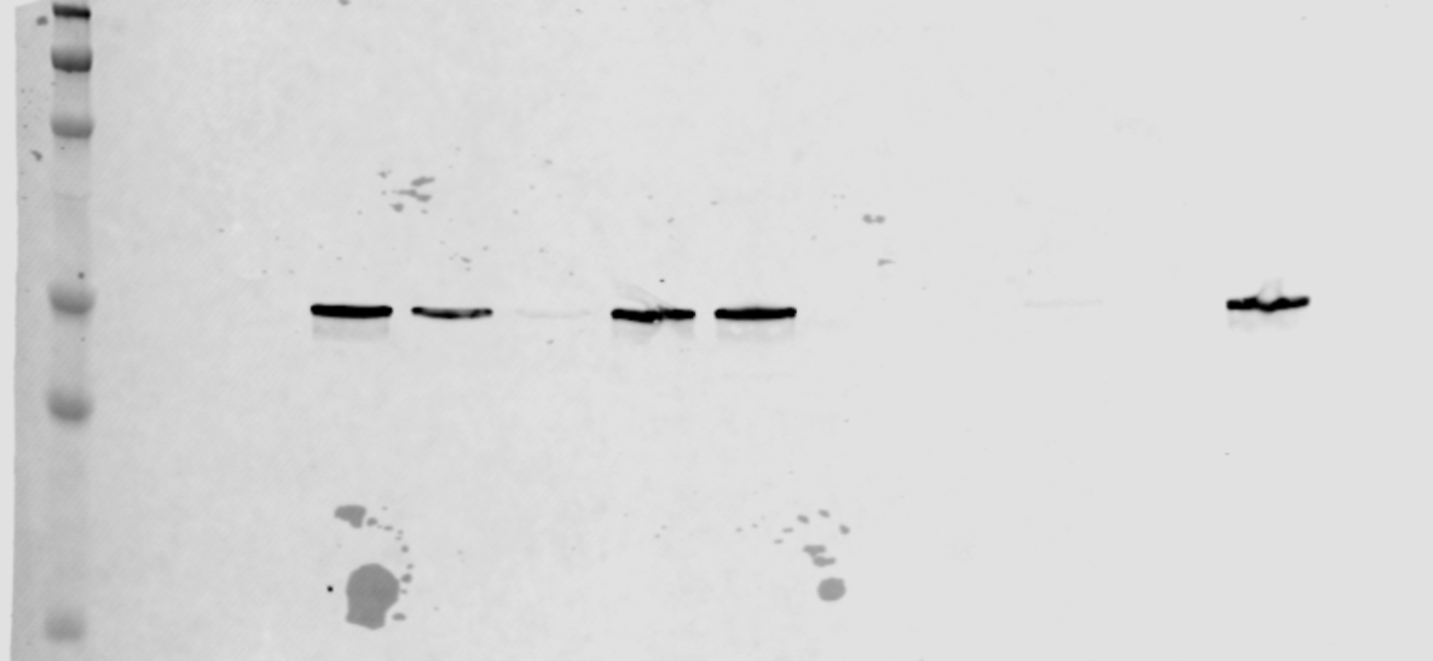


pHisNAMPT

1 2 3 4 5 6 7 8

**Detection of pHisNAMPT by western blotting**. Human NAMPT (20 μg/ml) was incubated with ATP (2 mM) (lanes 2-8) in TMD buffer for 10 min at 37°C. Also present were: 10 μM NAM (lane 4), 10 μM PRPP (lane 5), NAM + PRPP (lane 6), 10 μM NMN (lane 7), 10 μM PP (lane 8). Sample loaded in lane 2 was pre-heated at 95°C for 5 min after addition of the SDS-containing gel loading buffer. Samples were fractionated by SDS-PAGE, transferred to PVDF membranes, and probed with anti-1-pHis antibody. pHisNAMPT was detected with a LICOR Odyssey infrared imaging system. Source data are provided as a Source Data file.

b

*

*

#

#

&

**Supplementary Figure 8**

a

*

#

#

c

*

*

&

**NMNAT did not compromise the ability of SBI-797812 to stimulate NAMPT activity.** NAMPT (50 nM) was incubated with NAM (25 μM), PRPP 50 μM), ATP (2 mM) and (where indicated) SBI-797812 (5 μM), NMNAT (200 ng ml^-1^) or NMNAT + SBI-797812. NMN (panel a) and NAD^+^ (panel b) were assayed by LC-MS/MS. Panel c shows the sum of NMN and NAD^+^. Data are expressed as means ± s.d.; n=4 for all entries except (NAMPT + SBI-797812) which was n=3. *, p<0.05 vs. NAMPT; #, p<0.001 versus (NAMPT + SBI-797812; &, p<0.02 vs. (NAMPT + NMNAT). 1 way ANOVA with Tukey’s multiple comparisons test was used.

**Supplementary Figure 9**

*

#

$

*

#

**Human pyrophosphatase 1 (PPA1) did not compromise the ability of SBI-797812 to stimulate NAMPT activity.** NAMPT was incubated with NAM, PRPP, ATP and (where indicated) SBI-797812, PPA1 or SBI-797812 + PPA1. NMN was assayed using the fluorescence assay. Data are expressed as means ± s.d.; n=4. *, p<0.0001 versus NAMPT; #, p<0.0001 versus (NAMPT + SBI-797812); $, p<0.0001 versus (NAMPT + PPA1). 1 way ANOVA with Tukey’s multiple comparisons test was used. Source data are provided as a Source Data file.

**Supplementary Figure 10**

**

*

a.

b.

**SBI-797812 increased NMN and NAD+ in mouse primary myotubes.** Mouse primary myotubes were treated with vehicle or SBI-797812 (10 μM) for 4 h. NMN (panel a) and NAD^+^ (panel b) assayed by LC/MS/MS. Data shows means ± s.d.; n=6. *, p<0.002, **, p<0.0001. Two-tailed unpaired t test was used.

**Supplementary Figure 11**

a.

b.

**Plasma exposure after oral or i.p. dosing of SBI-797812 in mice.** Fasted male ICR mice were dosed with SBI-797812 via oral (10 mg kg^-1^, panel a) or i.p. (10 mg kg^-1^ panel b) administration. SBI-797812 was dosed as a 1 mg ml^-1^ solution in 10% DMSO / 10% Tween-80 / 80% water. Blood was drawn at 0, 0.25, 0.5, 1, 2, and 4 h, and plasma was prepared by centrifugation. Plasma levels of SBI-797812 were measured by mass spectrometry. Lines connect the mean values at each time point; n=3. Source data are provided as a Source Data file.

**Supplementary Figure 12**

**Tissue Levels of SBI-797812.** Liver, heart, kidney, gastrocnemius and quadriceps were harvested from mice at 2 h after i.p. dosing of SBI-797812 (20 mg kg^-1^). Tissues were lyophilized and processed. SBI-797812 levels were measured by LC-MS/MS and normalized to mg of dry powder weight. Data are expressed as means ± s.d.; n=3. Source data are provided as a Source Data file.

**Supplementary Table 1**. Chemical reagents, antibodies, proteins, cell lines, mice and assay kits used for this investigation.

| **Chemical Reagents** | **Source** | **Catalog #** |
| --- | --- | --- |
| Acetophenone | Sigma Aldrich (St. Louis, MO) | A10701 |
| ADP-HPD | Millipore Sigma | 118415 |
| ATP | Sigma Aldrich (St. Louis, MO) | A7699 |
| ^13^C_10_-^15^N_5_-ATP | Cambridge Isotope Laboratories (Cambridge, MA) | CNLM-4265-CA-20 |
| Adenosine-5’-tetraphosphate (Ap4) | Jena Bioscience | NU-1102S |
| Beuthanasia-D | Patterson Veterinary | 07-807-3963 |
| CHS-828 | Cayman Chemicals | 11021 |
| Dithiothreitol (DTT) | Sigma Aldrich (St. Louis, MO) | D0632 |
| DMSO | Thermo Scientific | 85190 |
| FK-866 | Santa Cruz Biotechnology | sc-205325A |
| Formic acid | Sigma Aldrich (St. Louis, MO) | 33015 |
| MgCl2 | Sigma Aldrich (St. Louis, MO) | M2393 |
| Nicotinamide (NAM) | Acros Organics | AC128271000 |
| D_4_-NAM | Cambridge Isotope Laboratories (Cambridge, MA) | DLM-6883-0.1 |
| NAM (^13^C_3_, ^15^N) | Cambridge Isotope Laboratories (Cambridge, MA) | CNLM-9757-0.001 |
| NAD^+^ | Sigma Aldrich (St. Louis, MO) | N7007 |
| NMN | Sigma Aldrich (St. Louis, MO) | N3501 |
| Olaparib | Cayman Chemicals (Ann Arbor MI) | 10621 |
| Perchloric acid (PCA) | Sigma Aldrich (St. Louis, MO) | 311421 |
| PRPP | Chem Cruz | SC-217240A |
| Protease inhibitor cocktail | Roche | 11873580001 |
| Pyrophosphate (PP) | Sigma Aldrich (St. Louis, MO) | 71501 |
| Trichostatin A | Sigma Aldrich (St. Louis, MO) | T8552 |
| Triphosphate (P3) | Sigma Aldrich (St. Louis, MO) | 72061 |
| **Antibodies** | **Source** | **Catalog #** |
| Acetyl-histone H4(K16) | Millipore Sigma | 07-329 |
| 1-pHis Clone: SC1-1 | Millipore Corp | MABS1330 |
| 3-pHis Clone: SC56-2 | Millipore Corp | MABS1352 |
| Goat Anti-Rabbit 800 | LI-COR | 926-32211 |
| Histone H4 | Cell Signaling | 13919 |
| PARP | Cell Signaling Technology | 9532 |
| PAR | Trevigen | 4336-APC-050 |
| **Proteins (Other)** | **Source** | **Catalog #** |
| Benzonase nuclease | Sigma Aldrich | E1014 |
| Human Pyrophosphatase 1 (PPA1) | R&D Systems | 6557-PP-010 |
| Pyrophosphatase, yeast | Roche (Indianapolis, IN), Sigma | 10108987001 |
| **Cell Lines** | **Source** | **Catalog #** |
| A549, human lung carcinoma | ATCC | CCL-185 |
| **Mice** | **Source** | **Catalog #** |
| C57BL/6J | The Jackson Laboratory | 000664 |
| **Assay Kits** | **Source** | **Catalog #** |
| NAD/NADH-Glo Assay | Promega Corp | G9071 |
| Pi ColorLock Gold Phosphate Detection System | Innova Biosciences | 303-0030 |

**Supplementary Table 2.** Custom PCR primers for amplification of human NMNAT1 cDNA

| Primer 1 | 5’-AAAAAACTCGAGTGT-CTTAGCTTCTGCAGTGTTTCTCT-3’ |
| --- | --- |
| Primer 2 | 5’- AAAAAACCATGGGCATGGAAAATTCCGAGAA-GACTGAAGTG-3’ |

**Supplementary Table 3.** Small molecule screening data

| **Category** | **Parameter** | **Description** |
| --- | --- | --- |
| Assay | Type of assay | *in vitro* biophysical |
|  | Target | NAMPT (Uniprot #43490) |
|  | Primary measurement | Differential scanning fluorimetry/protein thermal shift (DSF) with Sypro Orange |
|  | Key reagents | Sypro Orange (ThermoFisher #S6651), ATP (Sigma-Aldrich #A7699) |
|  | Assay protocol | See Methods section |
| Library | Library size | 57004 compounds |
|  | Library composition | Diversity library |
|  | Source | ChemBridge |
|  | Additional comments | http://www.chembridge.com/screening_libraries/diversity_libraries/diverset/ |
| Screen | Format | 384-well plate |
|  | Concentration(s) tested | 25 μM, 0.25% DMSO |
|  | Plate controls | 16 wells negative control (DMSO); 16 wells positive control (20 μM CHS-828) |
|  | Reagent/ compound dispensing system | ThermoFisher Multidrop Combi for reagents; Labcyte Echo 555 for compounds |
|  | Detection instrument and software | ThermoFisher ViiA7 and Protein Thermal Shift analysis software |
|  | Assay validation/QC | Z’ factor >= 0.5 |
|  | Correction factors | None |
|  | Normalization | None |
|  | Additional comments | https://www.sbpdiscovery.org/medical-discovery/drug-discovery/prebys-center-for-drug-discovery/overview |
| Post-HTS analysis | Hit criteria | Tm (calculated by slope-derivative peak) shift ≥ 1 degree C  OR Z-score ≥ 5 |
|  | Hit rate | 0.9% |
|  | Additional assay(s) | Triplicate-well single-concentration hit confirmation (PTS assay, 25 μM compound)  Triplicate-well single-concentration and dose-response profiling in NAMPT activity assay, NAD-GLO format (25 μM compound) |
|  | Confirmation of hit purity and structure | Select compounds were re-purchased from ChemBridge, and structure and purity were verified analytically |

**Supplementary Methods**

NAMPT activator chemistry. All reactions involving air and moisture-sensitive reagents and solvents were performed under a nitrogen atmosphere using standard chemical techniques. Anhydrous solvents were purchased and freshly used from Sigma-Aldrich or EMD Biosciences. All organic reagents were used as purchased. Analytical thin-layer chromatography was performed on Partisil K6F silica gel 60 Å, 250 μm. Microwave-assisted reactions were performed using a CEM Discover system. ^1^H chemical shifts are reported in δ values in ppm in the corresponding solvent. All solvents used for chromatography on the synthetic materials were Fisher Scientific HPLC grade, and H_2_O was Millipore Milli-Q PP filtered. All NMR spectra for the synthetic materials were recorded on a Bruker Avance II 400 instrument. LC-MS analysis of synthetic materials was performed with a Waters Autopurification system, which consisted of a 2767 sample manager, 2545 binary gradient module, system fluidics organizer, 2489 UV/Vis detector, and 3100 mass detector, all controlled with MassLynx software. A Sunfire Analytical C18 5 μm column (4.6 × 50 mm) and stepwise gradient [10% [(MeCN + 0.1% TFA) in (H_2_O + 0.1% TFA)] to 98% [(MeCN + 0.1% TFA) in (H_2_O + 0.1% TFA)] for 9 min] was used for analytical LC-MS of final compounds. The final compounds were purified as described. All synthesized final compounds were determined to be ≥ 95% pure by LC-MS and ^1^H NMR.

*Synthesis of 4-(3-(Pyridin-4-ylmethyl)ureido)benzamide*


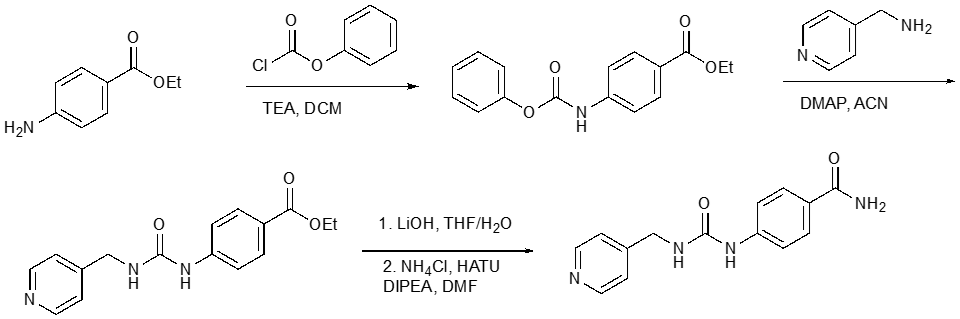


Step 1: To a solution of 4-amino-benzoic acid ethyl ester (500 mg, 3.03 mmol) in DCM (10 ml) was added phenyl carbonchloridate (570 mg, 3.63 mmol), followed by TEA (926 mg, 9.09 mmol). The resulting mixture was stirred at room temperature for 1 h. The reaction was monitored by TLC. The mixture was concentrated in *vacuum* to give a residue, which was purified by a silica gel column eluting with DCM to afford ethyl 4-((phenoxycarbonyl)amino)benzoate (826 mg, yield: 96%) as a white solid.

Step 2: To a solution of ethyl 4-((phenoxycarbonyl)amino)benzoate (826 mg, 2.89 mmol) in ACN (40 ml) was added *c*-pyridin-3-yl-methylamine (376 mg, 3.47 mmol), followed by DMAP (424 mg, 3.47 mmol). The resulting mixture was stirred at 80°C overnight. The reaction was monitored by LC-MS. The mixture was concentrated *in vacuum* to give a residue, which was purified by a reversed-phase column (5-95% ACN in H_2_O) to afford ethyl 4-(3-(pyridin-4-ylmethyl)ureido)benzoate (786 mg, yield: 91%) as a white solid. ^1^H NMR (400 MHz, DMSO-*d*_6_): *δ* = 9.18 (brs, 1H), 8.51 (dd, *J* = 4.4, 1.6 Hz, 2H), 7.84 (dd, *J* = 7.2, 1.6 Hz, 2H), 7.54 (dd, *J* = 7.2, 1.6 Hz, 2H), 7.29 (dd, *J* = 4.0, 1.6 Hz, 2H), 6.90 (t, *J* = 6.0 Hz, 1H), 4.34 (d, *J* = 6.0 Hz, 2H), 4.26 (q, *J* = 7.2 Hz, 2H), 1.30 (t, *J* = 7.2 Hz, 3H).

Step 3: To a solution of ethyl 4-(3-(pyridin-4-ylmethyl)ureido)benzoate (12.76 g, 42.6 mmol) in THF/H_2_O (100 ml + 30 ml) was added LiOH (5.37 g, 127.8 mmol). The resulting mixture was stirred at room temperature overnight. The reaction was monitored by TLC. THF was removed *in vacuum* to give an aqueous residue, which was acidified to pH = 4~5 with concentrated HCl. The solid precipitated from the mixture was filtered. The cake was washed with H_2_O (50 ml x 3) and dried in air to afford 4-(3-(pyridin-4-ylmethyl)ureido)benzoic acid (10.2 g, yield: 88%) as white solid.

Step 4: To a solution of 4-(3-(pyridin-4-ylmethyl)ureido)benzoic acid (55 mg, 0.20 mmol) in DMF (3 ml) was added HATU (116 mg, 0.31 mmol), DIPEA (131.0 mg, 1.01 mmol), and the mixture was stirred for 1 h at room temperature. Then NH_4_Cl (21.8 mg, 0.41 mmol) was added. The mixture was stirred overnight at room temperature. The mixture was concentrated under reduced pressure. The target (17 mg, yield: 31%) was obtained by prep-HPLC as a white solid. ^1^H NMR (400 MHz, CD_3_OD): δ = 8.47 (d, *J =* 5.2 Hz, 2H), 7.80 (d, *J =* 8.8 Hz, 2H), 7.49 (d, *J =* 9.6 Hz, 2H), 7.40 (d, *J =* 5.2 Hz, 2H), 4.46 (s, 2H). MS: m/z 271.3(M+H^+^).

*Synthesis of 1-[4-(8-oxa-3-aza-bicyclo[3.2.1]octane-3-sulfonyl)-phenyl]-3-pyridin-3-ylmethyl-urea (****SBI-796768; GNI-50****)*. GNI-50 was prepared as described previously ^1^

Step 1: To a solution of 8-oxa-3-aza-bicyclo[3.2.1]octane (100 mg, 0.668 mmol, HCl salt) in dry DCM (20 ml) was added 4-nitro-benzenesulfonyl chloride (296 mg, 1.34 mmol) and followed by TEA (202.8 mg, 2.0 mmol). The resulting mixture was stirred at room temperature for 2 h. The reaction was monitored by TLC. The mixture was concentrated *in vacuum* to give a residue which was purified by a silica gel column eluting with DCM/MeOH (60:1) to afford 3-(4-nitro-benzenesulfonyl)-8-oxa-3-aza-bicyclo[3.2.1]octane (150 mg, yield: 75%) as a white solid.

^1^H NMR (400 MHz, CDCl_3_): *δ* = 8.39 (dd, *J* = 7.2, 2.0 Hz, 2H), 7.92 (dd, *J* = 6.8, 2.0 Hz, 2H), 4.39 (s, 2H), 3.45 (d, *J* = 11.6 Hz, 2H), 2.27 (dd, *J* = 11.2, 2.0 Hz, 2H), 2.05-1.95 (m, 4H).

Step 2: To a solution of 3-(4-nitro-benzenesulfonyl)-8-oxa-3-aza-bicyclo[3.2.1]octane (100 mg, 0.335 mmol) in EtOAc (20 ml) was added Pd/C (20 mg, 20% wt). The resulting suspension was stirred at room temperature overnight. The reaction was monitored by LC-MS. Then Pd/C was filtered off and the filtrate was concentrated *in vacuum* to afford 4-(8-oxa-3-aza-bicyclo[3.2.1]octane-3-sulfonyl)-phenylamine (83 mg, yield: 92%) as a white solid.

^1^H NMR (400 MHz, DMSO-*d*_6_): *δ* = 7.32 (d, *J* = 8.8, 2H), 6.64 (d, *J* = 8.8 Hz, 2H), 6.07 (brs, 2H), 4.31 (s, 2H), 3.15 (d, *J* = 11.6 Hz, 2H), 2.37 (dd, *J* = 11.6, 2.0 Hz, 2H), 1.82-1.74 (m, 4H).

Step 3: To a solution of 4-(8-oxa-3-aza-bicyclo[3.2.1]octane-3-sulfonyl)-phenylamine (100 mg, 0.373 mmol) in DCM (10 ml) was added phenyl carbonchloridate (117 mg, 0.745 mmol) and followed by TEA (113 mg, 55.4 mmol). The resulting mixture was stirred at room temperature overnight. The reaction was monitored by TLC. The mixture was concentrated *in vacuum* to give a residue which was purified by silica gel column eluting with DCM/MeOH (60:1) to afford [4-(8-oxa-3-aza-bicyclo[3.2.1]octane-3-sulfonyl)-phenyl]-carbamic acid phenyl ester (132 mg, yield: 92%) as a white solid.

^1^H NMR (400 MHz, CDCl_3_): *δ* = 7.71 (d, *J* = 9.2 Hz, 2H), 6.64 (d, *J* = 8.8 Hz, 2H), 7.42 (t, *J* = 8.0 Hz, 2H), 7.27 (t, *J* = 7.2 Hz, 1H), 7.22-7.18 (m, 2H), 4.36 (s, 2H), 3.38 (d, *J* = 11.2 Hz, 2H), 2.63 (dd, *J* = 7.2, 2.0 Hz, 2H), 2.06-1.90 (m, 4H).

Step 4: To a solution of [4-(8-oxa-3-aza-bicyclo[3.2.1]octane-3-sulfonyl)-phenyl]-carbamic acid phenyl ester (120 mg, 0.309 mmol) in dioxane (8 ml) was added *c*-Pyridin-3-yl-methylamine (40 mg, 0.371 mmol) and followed by TEA (94 mg, 0.927 mmol). The resulting mixture was stirred at 80°C overnight. The reaction was monitored by LC-MS. The white solid precipitated from the reaction mixture was filtered. The cake was washed with dioxane (20 ml) and dried in air to afford 1-[4-(8-oxa-3-aza-bicyclo[3.2.1]octane-3-sulfonyl)-phenyl]-3-pyridin-3-ylmethyl-urea (76.4 mg, yield: 62%) as a white solid.

^1^H NMR (400 MHz, DMSO-*d*_6_): *δ* = 9.19 (brs, 1H), 8.53 (d, *J* = 1.6 Hz, 1H), 8.46 (d, *J* = 5.2 Hz, 1H), 7.71 (d, *J* = 8.0 Hz, 1H), 7.64 (d, *J* = 8.8 Hz, 2H), 7.56 (d, *J* = 8.8 Hz, 2H), 7.36 (dd, *J* = 8.0, 4.8 Hz, 1H), 6.91 (t, *J* = 6.0 Hz, 1H), 4.34 (d, *J* = 6.4 Hz, 2H), 4.32 (s, 2H), 3.21 (d, *J* = 10.8 Hz, 2H), 2.40 (d, *J* = 10.0 Hz, 2H), 1.84-1.73 (m, 4H). MS: m/z 403.1 (M+H^+^).

*Synthesis of 1-[4-(8-oxa-3-aza-bicyclo[3.2.1]octane-3-sulfonyl)-phenyl]-3-pyridin-2-ylmethyl-urea (****SBI-796950****).*

The title compound was prepared in a similar fashion to 1-[4-(8-oxa-3-aza-bicyclo[3.2.1]octane-3-sulfonyl)-phenyl]-3-pyridin-3-ylmethyl-urea (GNI-50).

^1^H NMR (400 MHz, DMSO-*d*_6_): *δ* = 9.35 (brs, 1H), 8.53 (d, *J* = 4.0 Hz, 1H), 7.78 (dt, *J* = 8.0, 2.0 Hz, 1H), 7.65 (d, *J* = 8.8 Hz, 1H), 7.64 (d, *J* = 8.8 Hz, 2H), 7.56 (d, *J* = 8.4 Hz, 2H), 7.35 (d, *J* = 7.6 Hz, 1H), 6.96 (t, *J* = 5.6 Hz, 1H), 4.43 (d, *J* = 6.0 Hz, 2H), 4.32 (s, 2H), 3.21 (d, *J* = 10.8 Hz, 2H), 2.41 (dd, *J* = 11.2, 2.0 Hz, 2H), 1.83-1.74 (m, 4H). MS: m/z 403.1 (M+H^+^).

1-[4-(8-Oxa-3-aza-bicyclo[3.2.1]octane-3-sulfonyl)-phenyl]-3-pyridin-4-ylmethyl-urea (**SBI-797812**).

The title compound was prepared as prepared in a similar fashion to 1-[4-(8-oxa-3-aza-bicyclo[3.2.1]octane-3-sulfonyl)-phenyl]-3-pyridin-3-ylmethyl-urea (GNI-50).

^1^H NMR (400 MHz, DMSO-*d*_6_): *δ* = 9.51 (brs, 1H), 8.74 (d, *J* = 6.0 Hz, 2H), 7.73 (d, *J* = 6.4 Hz, 2H), 7.65 (d, *J* = 9.2 Hz, 2H), 7.57 (d, *J* = 8.8 Hz, 2H), 7.17 (t, *J* = 5.6 Hz, 1H), 4.52 (d, *J* = 6.4 Hz, 2H), 4.32 (s, 2H), 3.21 (d, *J* = 11.2 Hz, 2H), 2.40 (dd, *J* = 11.2, 2.0 Hz, 2H), 1.84-1.73 (m, 4H). MS: m/z 403.1 (M+H^+^).

Synthesis of heavy isotope labeled NMN, NAD^+^ and NADH

*β-Nicotinamide Mononucleotide (NMN):* An oven-dried 12 ml vial was flushed with N_2_ and charged with nicotinamide riboside (as chloride salt, 386 mg, 1.33 mmol). Trimethyl phosphate (3.0 ml) was added at room temperature and the vial was placed under high vacuum until all volatile material evaporated (~2 min). The reaction mixture was then flushed with N_2_ and cooled to -5°C. Phosphorus oxychloride (495 μl, 5.31 mmol) was added the solution was stirred for 6.5 h after which H_2_^18^O (1.0 ml) was added and the stirring continued at -5°C overnight. The mixture was poured into MeOH (60 ml) containing NaOAc (1.0 g) and then directly injected onto a Biotage KP-NH (55 g) column. Elution at 35 ml min^-1^ with: 1) 5 column volumes (CV) of 0.1 M AcOH in MeOH; 2) 1 CV gradient to 0.1 M AcOH in H_2_O; 3) 5 CV of 0.1 M AcOH in H_2_O. The fractions containing the final product were combined, concentrated, and then lyophilized to obtain ^18^O_2_-β-NMN as a white powder (349 mg, 78% yield). ^1^H-NMR (500 MHz, D_2_O): δ 9.40 (d, *J* = 1.5 Hz, 1H), 9.22 (dd, *J* = 6.3, 1.4 Hz, 1H), 8.91 (dt, *J* = 8.2, 1.5 Hz, 1H), 8.23 (dd, *J* = 8.1, 6.3 Hz, 1H), 6.14 (d, *J* = 5.5 Hz, 1H), 4.57 (p, *J* = 2.4 Hz, 1H), 4.50 (t, *J* = 5.3 Hz, 1H), 4.38 (dd, *J* = 5.0, 2.5 Hz, 1H), 4.23 (ddd, *J* = 12.0, 4.3, 2.5 Hz, 1H), 4.07 (ddd, *J* = 12.1, 5.0, 2.1 Hz, 1H). ^13^C-NMR (125 MHz, D_2_O): δ 165.79, 145.97, 142.48, 139.84, 133.92, 128.51, 99.97, 87.44 (d, *J*_C-P_ = 8.8 Hz), 87.41, 77.73, 71.02, 64.13 (d, *J*_C-P_ = 4.9 Hz). LC-MS (ESI+, uncalibrated) for C_11_H_15_N_2_O_6_^18^O_2_P [M+H] expected = 339.07, found = 338.94.

*β-Nicotinamide Adenine Dinucleotide (NAD^+^):* A vial was charged with ^18^O_2_-β-Nicotinamide riboside (238 mg, 0.704 mmol) and formamide (3.5 ml, 0.2 M) at room temperature under a N_2_ atmosphere. To this solution was sequentially added adenosine 5-monophosphomorpholidate 4-morpholine-N,N-dicyclohexylcarboximidine (1.0 g, 1.41 mmol), *p*-toluenesulfonic acid monohydrate (402 mg, 2.11 mmol), anhydrous manganese(II) chloride (266 mg, 2.11 mmol), and pyridine (740 μl, 9.15 mmol). The resulting mixture was stirred overnight at room temperature under N_2_. The reaction completion was verified by the full consumption of adenosine monophosphate morpholidate as determined by LC-MS. The mixture was injected directly onto Biotage KP-NH column (55 g) and purified with the following eluents at 35 ml min^-1^: 1) 4 CV of 0.1 M AcOH in MeOH; 2) 4.5 CV of 0.1 M AcOH and 0.1 M NaOAc in MeOH; 3) 3 CV of 0.1 M AcOH in MeOH; 4) 1 CV gradient to 0.1 M AcOH in H_2_O; 5) 3 CV of 0.1 M AcOH in H_2_O; 6) 3 CV of 0.01 M AcOH in H_2_O; 7) 3 CV of 0.01 M AcOH and 0.15 M NaOAc in H_2_O. The fractions containing NAD^+^ were combined, concentrated, and then reconstituted with 10 ml of H_2_O and filtered through a cation exchange cartridge (SCX-2, 5 g, Biotage) to remove Mn^2+^. The cartridge was washed with 50 ml H_2_O to recover the metal free NAD^+^. The solution was concentrated, dissolved in 10 ml of H_2_O, and then injected directly onto a Biotage KP-NH column (55 g) and purified with the following eluents at 35 ml min^-1^: 1) 4.5 CV of 0.1 M AcOH in MeOH; 2) 1 CV gradient to 1.0 M AcOH in H_2_O; 3) 6 CV of 1.0 M AcOH in H_2_O. The fractions containing NAD^+^ were combined and concentrated. Acetic acid was removed from the sample by repetitive addition of H_2_O and evaporation (3 times) followed by lyophilization. The final product was obtained as a white solid (144 mg, 31% yield). ^1^H-NMR (500 MHz, D_2_O): δ 9.29 (s, 1H), 9.12 (d, *J* = 6.2 Hz, 1H), 8.79 (dt, *J* = 8.1, 1.4 Hz, 1H), 8.38 (s, 1H), 8.15 (dd, *J* = 8.1, 6.2 Hz, 1H), 8.12 (s, 1H), 6.04 (d, *J* = 5.5 Hz, 1H), 5.97 (d, *J* = 5.6 Hz, 1H), 4.66 (t, *J* = 5.4 Hz, 2H), 4.49 (t, *J* = 2.6 Hz, 1H), 4.43 (dt, *J* = 7.4, 5.1 Hz, 2H), 4.37 (dd, *J* = 5.0, 2.8 Hz, 1H), 4.30 (dq, *J* = 6.5, 3.1 Hz, 2H), 4.24 – 4.07 (m, 3H). ^13^C-NMR (125 MHz, D_2_O): δ 165.28 , 149.54 , 148.55 , 145.82 , 142.42 , 140.71 , 139.86 , 133.68 , 128.59 , 118.26 , 99.95 , 87.15 , 87.03 (d, *J*_C-P_ = 8.8 Hz), 83.88 (d, *J*_C-P_ = 8.8 Hz), 77.56 , 74.21 , 70.65 , 70.27 , 65.28 (d, *J*_C-P_ = 4.5 Hz), 64.90 (d, *J*_C-P_ = 5.0 Hz). LCMS (ESI+, uncalibrated) for C_21_H_27_N_7_O_12_^18^O_2_P_2_ [M+H] expected = 668.12, found = 668.00.

*β-Nicotinamide adenine dinucleotide, reduced diammonium salt (NADH*)**:** A vial was charged with NAD^+^ (45 mg, 0.067 mmol) and a N_2_-sparged aqueous solution of NaHCO_3_ (1.3%, 65 mg NaHCO_3_ in 5 ml H_2_O). Na_2_S_2_O_4_ (35 mg, 0.202 mmol) was then added and stirred under a N_2_ atmosphere at room temperature for 3 h. The solution was then quenched by bubbling air through the solution for 15 min. The solution was then purified via preparatory HPLC with a Hypercarb column (5 µM, 50 x 10 mm) using a gradient of 0.01 % NH_3_ in H_2_O and ACN at a 5 ml min^-1^ flow rate. Lyophilization of the fractions containing NADH gave a white solid (11.8 mg, 26% yield) which was stored < -20 °C. ^1^H-NMR (500 MHz, D_2_O): δ 8.42 (s, 1H), 8.17 (s, 1H), 6.87 (s, 1H), 6.05 (d, *J* = 5.5 Hz, 1H), 5.91 (d, *J* = 8.3 Hz, 1H), 4.80-4.70 (m, 2H), 4.64 (t, *J* = 4.9 Hz, 1H), 4.43 (t, *J* = 4.3 Hz, 1H), 4.30 (d, *J* = 1.8 Hz, 1H), 4.21 – 4.08 (m, 4H), 4.03 – 3.97 (m, 3H), 2.72 (d, *J* = 18.2 Hz, 1H), 2.60 (dd, *J* = 18.5, 3.6 Hz, 1H). LC-MS (ESI+, uncalibrated) for C_21_H_27_N_7_O_12_^18^O_2_P_2_ [M+H] expected = 670.14, found = 670.47.

Mass spectrometry to detect NAMPT products.

NAM, NAD^+^, 1-MeNAM, ADP and Ap4.  *Preparation of Standards for LC-MS/MS****.*** Individual stock solutions of standards were prepared by dissolving each nucleotide in 0.1% formic acid at concentrations of 250 mM for NAM, 50 mM for NAD^+^, 1.25 mM for NMN, 50 mM for 1 Me-NAM, and 250 mM for ADP. A combined calibration stock solution was prepared by mixing aliquots of each stock solution and diluting in 0.1% formic acid. The working calibration curve was prepared by serial dilution of the combined calibration stock solution in 0.1% formic acid. The working calibration curve concentrations were 2.5, 5, 10, 25, 50, 100, 250, 500, 1000, and 2000 µM for NAM and NAD^+^. NMN working calibration solutions were 0.025, 0.05, 0.1, 0.025, 0.5, 1, 2.5, 5, 10, and 20 µM. 1-MeNAM working calibration solutions were 1.25, 2.5, 5, 12.5, 25, 50, 125, 250, 500, and 1000 µM. ADP and Ap4 working calibration solutions were 6.25, 12.5, 25, 62.5, 125, 250, 625, 1250, 2500, and 5000 µM. Then, calibration curve solutions for LC-MS/MS were prepared by spiking 10 µl aliquots of each working calibration solution into a mixture of 90 µl of 0.5 M PCA and 100 µl of 1 M ammonium formate. This resulted in final calibration solutions of 0.25, 0.5, 1, 2.5, 5, 10, 25, 50, 100, and 200 µM for NAD^+^ and NAM, 0.0025, 0.005, 0.01, 0.025, 0.05, 0.1, 0.25, 0.5, 1, and 2 µM for NMN, 0.125, 0.25, 0.5, 1.25, 2.5, 5, 12.5, 25, 50, and 100 µM for 1-MeNAM, and 0.625, 1.25, 2.5, 6.25, 12.5, 25, 62.5, 125, 250, and 500 µM for ADP and Ap4. Individual stock IS solutions were prepared by dissolving each isotopically- labelled nucleotide in 0.1% formic acid. Stock concentrations were 20 mM for ^18^O_2_-NAD^+^, 0.25 mM for ^18^O_2_-NMN, 50 mM for d_4_-NAM, 10 mM for 1-Me-d_3_-NAM, and 10 mM for (^13^C_10_, ^15^N_5_)-ATP. Aliquots of these solutions were combined to prepare a working IS working solution of 100 µM for ^18^O_2_-NAD^+^, 1.25 µM for ^18^O_2_-NMN, 250 µM for d_4_-NAM, 50 μM for 1-Me-d_3_-NAM, and 50 µM for (^13^C_10_, ^15^N_5_)-ATP. Then, a 10 µl aliquot of the working IS solution was spiked into each final calibration solution. *Extraction of Nucleotides***.** NAMPT assay sample (100 μl) was quenched with equal volume 1 M PCA and spiked with a 10 μl mixture of isotopic labeled IS. 1 M ammonium formate (100 μl) was added to adjust the pH to ~4. Samples were vortexed and centrifuged at 18,000 x g for 5 min at 10°C. The samples were passed through an AcroPrep Advance 3K Omega Filter Plate (Pall Corporation) by centrifugation at 3500 x g for 60 min prior to LC-MS/MS analysis. *Liquid Chromatography***.** Metabolites were separated on a 2.1 x 50 mm, 3 μm Thermo Scientific Hypercarb column (T = 30°C) using a Dionex Ultimate 3000 UHPLC. The step gradient was 98% A (10 mM ammonium acetate, pH 9.5) and 2% B (ACN) to 64 % A and 36% B over 6.3 min. The step gradient began at 2% B (0.6 ml min^-1^ flow rate) from 0-0.45 min, was increased from 2% to 36% B (0.6 ml min^-1^ flow rate) from 0.45-6.3 min, was increased from 36% to 95% B (0.8 ml min^-1^ flow rate) from 6.3-6.4 min, and was held until 8.4 min. Re-equilibration was performed at 2% B from 8.4-8.5 min (0.7 ml min^-1^ flow rate) and was held until 11.5 min. The flow returned to 0.6 ml min^-1^ at 11.6 min and was held until 11.7 min. Samples were injected (5 µl) on a LEAP CTC PAL autosampler maintained at 5°C. *Mass Spectrometry***.** The instrument method was created with Xcalibur 3.0 and data acquisition was performed by TraceFinder 3.2. Quantitation of pyridine nucleotides was achieved using single reaction monitoring (SRM) on a Thermo Scientific Quantiva triple quadrupole mass spectrometer (Thermo Scientific). The mass spectrometer was operated in positive ion mode using electrospray ionization with an ESI capillary voltage of 3500 V. The ion transfer tube temperature was 350 °C and vaporizer temperature was 350°C. The ESI source sheath gas was set to 40, the auxiliary gas was set to 10, and the sweep gas was set to 1. The mass spectrometer was operated with a mass resolution of 0.7 Da, a cycle time of 0.3 s, and nitrogen collision gas of 1.5 mTorr for the generation and detection of product ions of each nucleotide. SRM transitions were 123.1 🡪 80.1 for NAM, 127.1 🡪 84.1 for d_4_-NAM, 137.2 🡪 94.1 for 1-MeNAM, 141.2 🡪 98.1 for 1-Me-d_3_-NAM, 335.2 🡪 123.1 for NMN, 339.2 🡪 123.1 for ^18^O_2_-NMN, 428.0 🡪 136.1 for ADP, 508.1 🡪 136.1 for ATP, 523.1 🡪 146.1 for ^13^C_10_-^15^N_5_-ATP, 664.2 🡪 428.1 for NAD^+^, 668.2 🡪 432.1 for ^18^O_2_-NAD^+^, and 588.0 🡪 136.1 for Ap4. Collision energies to produce product ions ranged from 16-46 V with RF lens values ranging from 43-85 V. Raw data was processed using Xcalibur 3.0. Calibration curves (R^2^ = 0.99 or greater) were either fitted with a linear or a quadratic curve with a 1/X or 1/X^2^ weighting.

NADH and NADPH. Individual stock solutions of NADH and NADPH standards (12.5 mM each) were prepared in 10 mM NaOH. Aliquots of each reduced nucleotide stock solution were combined to prepare a calibration stock solution (0.5 mM NADH and NADPH in 10 mM NaOH). A working calibration curve was prepared in 10 mM NaOH by serial dilution of the combined calibration stock solution to yield concentrations of 0.625, 1.25, 2.5, 5, 10, 25, 50, 100, and 500 µM for NADH and NADPH. Ten µl aliquots of each working calibration curve solution were added to vials containing 190 µl of 50:50 0.1 M NaOH/MeOH. The final calibration solutions for LC-MS/MS were 0.031, 0.062, 0.125, 0.25, 0.5, 1.25, 2.5, 5.0, 12.5, and 25 µM for NADH and NADPH. A stock solution of 50 mM ^18^O_2_-NADH in 10 mM NaOH was prepared and diluted to make a 50 μM solution in 10 mM NaOH. A 10 µl aliquot of NADH IS working solution was spiked into each final calibration solution followed by vortexing. *Extraction of Nucleotides****.*** Cells were homogenized in 500 μl 50:50 0.1 M NaOH/MeOH using a Precellys bead-based homogenizing system attached to a Crylolys cooling system. A 200 µl aliquot of the homogenate was spiked with a 10 µl aliquot of an isotopically-labeled NADH IS solution. Samples were vortexed thoroughly and centrifuged at 18,000 x g for 5 min at 10°C. Then, samples were transferred to a 96-well AcroPrep Advance 3K Omega Filter Plate (Pall Corporation), placed over a 96-well plate, and centrifuged at 3500 x g and 10°C for 60 min or until the volume of filtrate was adequate for LC-MS/MS analysis. *Liquid Chromatography***.** NADH and NADPH were separated on a HSST3 Waters Acquity UPLC column (2.1 mm x 150 mm, 1.8 µm). The mobile phase gradient was 100% A (5 mM ammonium acetate, pH 6) and 0% B (ACN) to 45% A and 55% B over 4.9 min. The gradient began at 0% B (0.5 ml min^-1^ flow rate) from 0-3.0 min, was increased 0% to 55% B (0.5 ml min^-1^ flow rate) from 3.0-4.9 min, was increased 55% to 90% B (0.5 ml min^-1^ flow rate) from 4.9-5.0 min, and was held until 8.0 min. Re-equilibration was performed at 0% B from 8.0-8.1 min (0.5 ml min^-1^ flow rate) and was held until 14.1 min. The samples were injected (5 µl) using a LEAP CTC PAL autosampler maintained at 5°C. *Mass Spectrometry****.*** The instrument method was created with Xcalibur 3.0 and data acquisition was performed with TraceFinder 3.2. NADH and NADPH were quantified by SRM using a Thermo Scientific Quantiva triple quadrupole mass spectrometer operated in positive ion mode using electrospray ionization with an ESI capillary voltage of 3500V. The ion transfer tube temperature was 350°C and vaporizer temperature was 300°C. The ESI source sheath gas was set to 40, the auxiliary gas was set to 10, and the sweep gas was set to 1. The mass spectrometer was operated with a mass resolution of 0.7 Da, a cycle time of 0.3 s, and nitrogen collision gas of 1.5 mTorr for the generation and detection of product ions of NADH and NADPH. SRM transitions were 666.2 🡪 649.2 for NADH, 670.2 🡪 653.2 for ^18^O_2_-NADH, and 746.2 🡪 729.2 for NADPH. The collision energy to produce product ions was 25 V with RF lens values ranging from 80-85. *Data Processing*. Raw data was processed using Xcalibur 3.0. Calibration curves (R^2^ = 0.99 or greater) were either fitted with a linear or a quadratic curve with a 1/X or 1/X^2^ weighting.

Triphosphate (P3). A stock solution of P3 (100 μM) was prepared in LC-MS grade H_2_O. The working calibration curve was prepared by spiking the stock solution in 50 µl of TMD buffer for a final P3 concentrations of 5, 10, 15, 20 and 25 µM. Experimental samples were transferred to a 96-well AcroPrep Advance 3K Omega filter plate, placed over a 96-well plate, and centrifuged at 3500 x g and 10°C for 60 min or until the volume of filtrate was adequate for LC-MS/MS analysis. The P3 levels were determined using Waters Acquity UPLC BEH C18, column (2.1X150mm, 1.7 µM) (T = 40°C) and a Dionex Ultimate 3000 UHPLC. The mobile phase gradient was 100 % A (10 mM tributylamine+15 mM acetic acid) and 0% B (ACN) to 2% A and 98 % B over 4.1 min. The gradient began at 100% A (0.4 ml min^-1^ flow rate) from 0- 1.0 min, was increased 0% to 98% B (0.4 ml min^-1^ flow rate) from 1.0-4.1 min. Re-equilibration was performed at 0% B (0.4 ml min^-1^ flow rate) from 4.1 to 7.1 min and was held until 11.5 min. The samples were injected (10 µl) using a LEAP CTC PAL autosampler maintained at 5°C. The instrument method was created with Xcalibur 3.0 and data acquisition was performed with TraceFinder 3.2. Quantitation of P3 was achieved by SRM using a Thermo Scientific Quantiva triple quadrupole mass spectrometer. The mass spectrometer was operated in negative ion mode using electrospray ionization with an ESI capillary voltage of 2500 V. The ion transfer tube temperature was 350°C and vaporizer temperature was 350 °C. The ESI source sheath gas was set to 40, the auxiliary gas was set to 10, and the sweep gas was set to 1. The mass spectrometer was operated with a mass resolution of 0.7 Da, a cycle time of 0.3 s, and nitrogen collision gas pressure of 1.5 mTorr for the generation and detection of product ions of P3. SRM transitions was 256.9 🡪 158.9 for P3. Collision energies to produce product ions was 17 V with RF lens values ranging from 64V. P3 raw data was processed using Xcalibur 3.0. The P3 calibration curve was plotted using the raw area counts from known working calibration solutions.

Sn2 transition structure for the attack of PP on pHisNAMPT.

The transition structure was explored using the DFT method B3LYP/6-31G* as implemented in Gaussian 09.2 Model was built from an overlay of the active site of the relevant crystal structures of human NAMPT complexed with NMN and PP (PDB:3DHF) and human NAMPT complexed with benzamide and PRPP (PDB: 3DKL); residues within contact distance of the PP/pHis in the crystal structures were included in the model for electronic structure calculations: Asp313, Asp 279, pHis247, PP, 2 Mg, and water (Asp residues were modeled as acetate, pHis as the imidazole/phosphate, and the water and 2 Mg were necessary for stabilizing the PP group). A transition structure was located without any geometric restrictions by performing an opt=(modredun,loose) optimization of an input structure with the key bond-forming and bond-breaking distances held constant. This was followed by a transition structure search using the keyword opt=(calcfc,ts,noeigentest,nofreeze) freq. The calculated transition structure was located as a stationary point and possesses only one imaginary frequency, which corresponds to the SN2 reaction coordinate.

Energies:

SCF Done: E(RB3LYP) = -3320.23502476

Enthalpy 0K = -3319.836302

Enthalpy 298K = -3319.789841

Free Energy 298K = -3319.918999

Nimag = 1 (-173.4158 cm-1)

Coordinates:


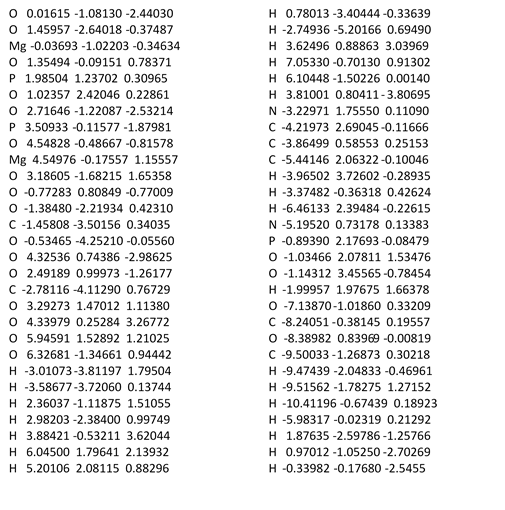


**Supplementary References**

1 Zheng, X. *et al.* Structure-based identification of ureas as novel nicotinamide phosphoribosyltransferase (Nampt) inhibitors. *J Med Chem* **56**, 4921-4937, doi:10.1021/jm400186h (2013).

2 Gaussian 09, Revision A.02, M. J. Frisch, G. W. Trucks, H. B. Schlegel, G. E. Scuseria, M. A. Robb, J. R. Cheeseman, G. Scalmani, V. Barone, G. A. Petersson, H. Nakatsuji, X. Li, M. Caricato, A. Marenich, J. Bloino, B. G. Janesko, R. Gomperts, B. Mennucci, H. P. Hratchian, J. V. Ortiz, A. F. Izmaylov, J. L. Sonnenberg, D. Williams-Young, F. Ding, F. Lipparini, F. Egidi, J. Goings, B. Peng, A. Petrone, T. Henderson, D. Ranasinghe, V. G. Zakrzewski, J. Gao, N. Rega, G. Zheng, W. Liang, M. Hada, M. Ehara, K. Toyota, R. Fukuda, J. Hasegawa, M. Ishida, T. Nakajima, Y. Honda, O. Kitao, H. Nakai, T. Vreven, K. Throssell, J. A. Montgomery, Jr., J. E. Peralta, F. Ogliaro, M. Bearpark, J. J. Heyd, E. Brothers, K. N. Kudin, V. N. Staroverov, T. Keith, R. Kobayashi, J. Normand, K. Raghavachari, A. Rendell, J. C. Burant, S. S. Iyengar, J. Tomasi, M. Cossi, J. M. Millam, M. Klene, C. Adamo, R. Cammi, J. W. Ochterski, R. L. Martin, K. Morokuma, O. Farkas, J. B. Foresman, and D. J. Fox, Gaussian, Inc., Wallingford CT, 2016.
