# Supplementary material for: Boosting NAD+ with a small molecule that activates NAMPT
Source: Nat Commun. 2019 Jul 19;10:3241. doi: 10.1038/s41467-019-11078-z (PMC6642140; doi:10.1038/s41467-019-11078-z)
Supplement: Supplementary file 3 — Reporting Summary [file 41467_2019_11078_MOESM3_ESM.pdf]

nature research

Corresponding author(s): Stephen J. Gardell, Anthony B. Pinkerton

Last updated by author(s): May 29, 2019

Reporting Summary

Nature Research wishes to improve the reproducibility of the work that we publish. This form provides structure for consistency and transparency in reporting. For further information on Nature Research policies, see Authors & Referees and the Editorial Policy Checklist.

Statistics

For all statistical analyses, confirm that the following items are present in the figure legend, table legend, main text, or Methods section.

n/a	Confirmed	
☐	☒	The exact sample size (*n*) for each experimental group/condition, given as a discrete number and unit of measurement
☐	☒	A statement on whether measurements were taken from distinct samples or whether the same sample was measured repeatedly
☐	☒	The statistical test(s) used AND whether they are one- or two-sided<br>*Only common tests should be described solely by name; describe more complex techniques in the Methods section.*
☒	☐	A description of all covariates tested
☐	☒	A description of any assumptions or corrections, such as tests of normality and adjustment for multiple comparisons
☐	☒	A full description of the statistical parameters including central tendency (e.g. means) or other basic estimates (e.g. regression coefficient) AND variation (e.g. standard deviation) or associated estimates of uncertainty (e.g. confidence intervals)
☐	☒	For null hypothesis testing, the test statistic (e.g. *F*, *t*, *r*) with confidence intervals, effect sizes, degrees of freedom and *P* value noted<br>*Give P values as exact values whenever suitable.*
☒	☐	For Bayesian analysis, information on the choice of priors and Markov chain Monte Carlo settings
☒	☐	For hierarchical and complex designs, identification of the appropriate level for tests and full reporting of outcomes
☒	☐	Estimates of effect sizes (e.g. Cohen's *d*, Pearson's *r*), indicating how they were calculated

Our web collection on statistics for biologists contains articles on many of the points above.

Software and code

Policy information about availability of computer code

Data collection	QuantStudio Real-time PCR System v.1.2 (Thermo Fisher Scientific) used for PTS HTS; PHERAstar version 5.41 (BMGF Labtech) used for NMN assay in HTS format; SoftMax Pro software (Molecular Devices) used for 96-well plate reader data; Xcalibur software version 3.0 (Thermo Scientific) used for mass spectrometer data
Data analysis	Applied Biosystems Protein Thermal Shift analysis software v.1.2 (Thermo Fisher Scientific); CBIS software (Cheminnovation Software Inc.) used for NMN assay in HTS format; GraphPad Prism version 6.0 (GraphPad Software, Inc); Xcalibur software version 3.0 (Thermo Scientific) used for mass spectrometer data. Gaussian 09 software used for transition state modeling.,

For manuscripts utilizing custom algorithms or software that are central to the research but not yet described in published literature, software must be made available to editors/reviewers. We strongly encourage code deposition in a community repository (e.g. GitHub). See the Nature Research guidelines for submitting code & software for further information.

Data

Policy information about availability of data

All manuscripts must include a data availability statement. This statement should provide the following information, where applicable:

- Accession codes, unique identifiers, or web links for publicly available datasets
- A list of figures that have associated raw data
- A description of any restrictions on data availability

All data supporting the findings of this study are available from the corresponding author (SJG) upon request

Field-specific reporting

Please select the one below that is the best fit for your research. If you are not sure, read the appropriate sections before making your selection.

☒ Life sciences ☐ Behavioural & social sciences ☐ Ecological, evolutionary & environmental sciences

For a reference copy of the document with all sections, see nature.com/documents/nr-reporting-summary-flat.pdf

Life sciences study design

All studies must disclose on these points even when the disclosure is negative.

Sample size	Sample sizes were chosen based on studies with similar experimental design and known variability of the assay
Data exclusions	No data was excluded
Replication	All of the data was repeated at least twice and the findings were reliably reproduced
Randomization	Mice were randomly allocated to the distinct groups
Blinding	Individuals performing the mass spectrometry assays were blinded to the identity of the samples

Reporting for specific materials, systems and methods

We require information from authors about some types of materials, experimental systems and methods used in many studies. Here, indicate whether each material, system or method listed is relevant to your study. If you are not sure if a list item applies to your research, read the appropriate section before selecting a response.

Materials & experimental systems

n/a	Involved in the study	
☐	☒	Antibodies
☐	☒	Eukaryotic cell lines
☒	☐	Palaeontology
☐	☒	Animals and other organisms
☐	☒	Human research participants
☒	☐	Clinical data

Methods

n/a	Involved in the study	
☒	☐	ChIP-seq
☒	☐	Flow cytometry
☒	☐	MRI-based neuroimaging

Antibodies

Antibodies used	Anti-N1-phosphohistidine rabbit monoclonal antibody, EMD Millipore, Cat# MABS1330, clone SC1-1, Lot # 2912011; Anti-N3-phosphohistidine rabbit monoclonal antibody, EMD Millipore, Cat# MABS1352, clone SC56-2, Lot#2860589; Anti-PARP rabbit monoclonal antibody, Cell Signaling Technologies, Cat# 9532, clone 46D11, Lot# 9 ; Anti-PAR rabbit polyclonal antibody, Trevigen, Cat#4336-APC, Lot#34180k15; Anti-Histone H4 rabbit monoclonal antibody, Cell Signaling, Cat# 13919, clone D2X4V, Lot#3; Anti-acetyl-histone H4(Lys16) rabbit polyclonal antibody, EMD Millipore, Cat#07-329, Lot#3126040. IRDye 800CW Goat (polyclonal) anti-rabbit IgG (H+L), LI-COR, Cat#926-32211.
Validation	The data sheets supplied by the highly-reputable commercial vendors amply supports the claimed epitope reactivities.

Eukaryotic cell lines

Policy information about cell lines

Cell line source(s)	A549 human lung carcinoma cell line was obtained from the ATCC (Manassas, Virginia). Human and mouse primary myotubes were differentiated from myoblasts isolated from human and mouse skeletal muscle specimens, respectively.
Authentication	A549 cells were not further authenticated. Myotubes exhibit a classical multi-nucleated tubular appearance that will on occasion display cellular contraction. Hence, the cellular phenotype of the myotubes (both human and mouse-derived) is serves as the basis for a definitive identification.
Mycoplasma contamination	All cell lines were tested negative for mycoplasma contamination
Commonly misidentified lines (See ICLAC register)	No commonly misidentified cell lines were used

Animals and other organisms

Policy information about studies involving animals; ARRIVE guidelines recommended for reporting animal research

Laboratory animals	M. musculus, C57BL/6J male, 8 weeks of age; puchased from the Jackson Laboratories
Wild animals	This study did not involve wild animals
Field-collected samples	This study did not involve field-collected samples
Ethics oversight	All animal studies and procedures were approved by the Institutional Animal Care and Use Committee at Sanford Burnham Prebys in Orlando FL (protocol # 2016-0136).

Note that full information on the approval of the study protocol must also be provided in the manuscript.

Human research participants

Policy information about studies involving human research participants

Population characteristics	The human muscle cells (myobloasts) were derived from both male and female healthy volunteers, aged 18-65. No genotyping was performed. No medical diagnosis was made since these cells were derived from healthy volunteers.
Recruitment	After a telephone screen to determine if they met criteria, research participants visited the research clinic at the Translational Research Institute for Metabolism and Diabetes at Advent Health in Orlando, FL. Potential participants were briefed on th study by study site personnel, reviewed an Informed Consent via an IRB governed process and provided witnessed written informed consent.
Ethics oversight	The IRB of record is the Advent health IRB which is accredited by the AAHRPP. Fouad Hajjar, MD serves as the IRB Chairman. FWA IRB00000842. Registered at Clinical Trials.gov as NCT02226640

Note that full information on the approval of the study protocol must also be provided in the manuscript.
